# Ring of resistance: Circular RNA Os-CircANK sequesters miR398b to counter rice blast resistance

**DOI:** 10.1093/plcell/koaf107

**Published:** 2025-05-05

**Authors:** Pei Qin Ng

**Affiliations:** Assistant Features Editor, The Plant Cell, American Society of Plant Biologists; Department of Plant Sciences, University of Cambridge, Cambridge CB2 3EA, UK

Circular RNAs are noncoding, single-stranded RNA molecules in which the downstream splice-donor site is covalently linked to the upstream donor site (Kristensen et al. 2019). In humans, circular RNA plays essential roles in cancer progression and serves as a biomarker in disease diagnostics (Xia et al. 2017; Chu et al. 2022). In plants, circular RNAs are abundant and show different characteristics than their animal counterparts, including different biogenesis processes (Zhang et al. 2020). In rice, circular RNAs originate from young parental genes, suggesting that these noncoding RNAs emerged relatively recently in plant evolution (Zhang et al. 2020; Chu et al. 2022). However, the function of these plant circular RNAs remains underexplored in the context of plant disease resistance.

In contrast, the role of RNA silencing via small RNAs (ranging from 21 to 26 nt) in plant immunity is well-established (Baulcombe 2004; Li et al. 2022). MicroRNAs, for example, mediate key plant processes, including development and stress regulation, with microRNA 398b (miR398b) being a regulator of stress and disease response (Li et al. 2022). Recent work by **Xiaohui Liu and colleagues** (Liu et al. 2025) shows that the circular RNA Os-CircANK lowers the ability of rice plants to combat the incoming infection via “sponging” miR398b. Their work unravels a novel regulation mechanism for counteracting disease resistance in rice.

To investigate the putative role of circular RNAs in disease resistance, Liu and colleagues identified Os-circANK, a circular RNA found to be downregulated in blight-infected rice. Next, the team generated Os-circANK knockdown (Os-circANK_KD_) and overexpressing (Os-circANK_OE_) plants and compared these with the wild-type rice plants. Infection assays with *Xanthomonas oryzae pv. oryzae* (*Xoo*), the main causal agent of blight disease in rice, showed that Os-circANK overexpressing plants (Os-circANK_OE_) displayed a more severe disease phenotype compared to wild-type or knockdown plants. Using a combination of computational predictions, dual luciferase assay, and RNA immunoprecipitations, miR398b was identified and validated as the target of Os-circANK. Fluorescent in-situ hybridization showed coexpression of miRNA398b and Os-circANK in the cytosol, supporting the role of Os-circANK as a competing endogenous RNA. These findings suggest that the depletion of miR398b is due to miRNA398b binding to Os-circANK, which prevents miR398b from binding to its target transcripts.

miRNA398b was found to be highly enriched in Os-circANK_OE_ and reduced in Os-circANK_KD_. plants. To investigate the interaction between Os-circANK and miR398b in more detail, Liu and colleagues further quantified the transcript levels of miR893b target genes, many of which regulate the production of reactive oxygen species. In Os-circANK_OE_ rice plants, these transcripts are no longer suppressed by miRNA398b, thereby impeding reactive oxygen species generation and leading to decreased resistance to *Xoo* infection (Figure). miR398b-overexpressing rice plants showed stronger disease resistance, with Os-circANK_KD_ lines showing similar effects. Interestingly, quantitative real-time PCR revealed that miR398b was highly expressed in *Xoo*-infected rice, while the transcript levels of its target genes were downregulated, further consolidating Os-circANK as the negative regulator of rice blight disease resistance.

**Figure.**
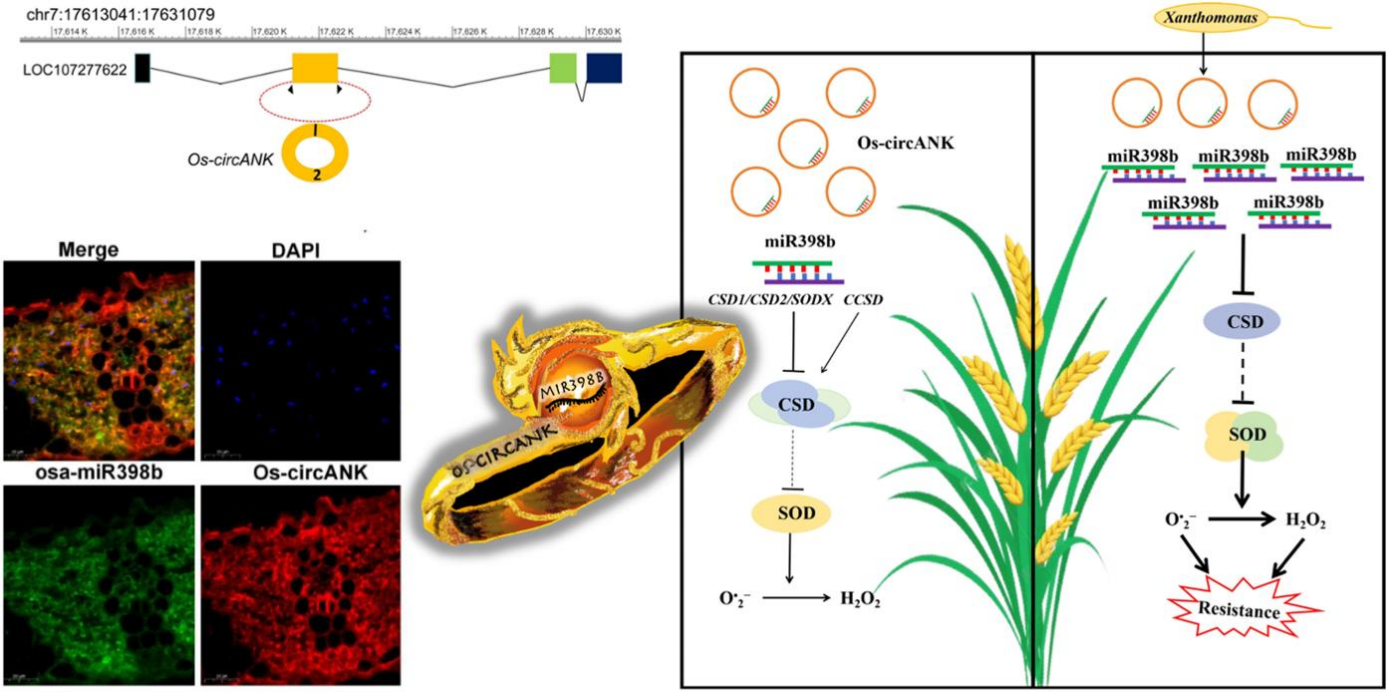
Rice Osa-miR398b is a sponge target of Os-circANK. Locus of Os-circANK on chromosome 7 (top left). Coexpression of Os-circANK and miRNA398b in the cytoplasm of rice plant cells indicates that Os-circANK can act as a competing endogenous RNA (ceRNA) (bottom left). Graphical representation of Os-circANK as the “ring” sequestering miR398b, which is responsible for infection response in plants (center). Proposed mechanism of Os-circANK (right). Under normal circumstances, rice plants maintain a basal level expression of Os-circANK and miR398b. Upon bacterial infection, a decrease in Os-circANK leads to the rise of miR398b, suppressing CSD genes and promoting the action of SOD, which enhances resistance against blight disease. Adapted from Liu et al. (2025), Figures 1A, 4G, 7.

In summary, the work of Liu et al. (2025) not only sheds light on the direct interaction of a circular RNA with a miRNA and its role in plant disease resistance but also presents a technique for studying circular RNAs. This work expands our knowledge of circular RNAs in plants and opens the door to their use as a tool to improve crop disease resistance.

## Recent related articles in *The Plant Cell*

- Attallah et al. (2025) review the roles of long and small ncRNAs in plant biology, highlighting their application as powerful tools in agricultural biotechnology.

## Data Availability

No new data were generated or analysed in support of this article.
